# Effect of Aerobic-Based Exercise on Psychological Well-Being and Quality of Life Among Older People: A Middle East Study

**DOI:** 10.3389/fpubh.2021.764044

**Published:** 2021-12-06

**Authors:** Amir Shams, Hadi Nobari, José Afonso, Hamed Abbasi, Elena Mainer-Pardos, Jorge Pérez-Gómez, Mahdi Bayati, Alireza Bahrami, Lara Carneiro

**Affiliations:** ^1^Department of Motor Behavior, Sport Sciences Research Institute, Tehran, Iran; ^2^Department of Physical Education and Sports, University of Granada, Granada, Spain; ^3^HEME Research Group, Faculty of Sport Sciences, University of Extremadura, Cáceres, Spain; ^4^Department of Exercise Physiology, Faculty of Educational Sciences and Psychology, University of Mohaghegh Ardabili, Ardabil, Iran; ^5^Centre for Research, Education, Innovation and Intervention in Sport (CIFI2D), Faculty of Sport of the University of Porto (FADEUP), Porto, Portugal; ^6^Department of Sport Injuries and Corrective Exercises, Sport Sciences Research Institute, Tehran, Iran; ^7^Health Sciences Faculty, Universidad San Jorge, Zaragoza, Spain; ^8^Department of Exercise Physiology, Sport Sciences Research Institute, Tehran, Iran; ^9^Department of Motor Behavior and Sport Psychology, Faculty of Sport Sciences, Arak University, Arak, Iran; ^10^Department of Sport and Physical Education, University of Maia (ISMAI), Maia, Portugal; ^11^Research Centre in Sports Sciences, Health Sciences and Human Development, CIDESD, GERON Research Community, Vila Real, Portugal

**Keywords:** cardiovascular fitness, mental health, old people, physical fitness, dose-response

## Abstract

The aimed to evaluate the effects of low and moderate-intensity aerobic exercise on psychological well-being (PWB) and quality of life (QoL) among older people. Forty-five male Iranian adults aged 65–80 years were selected according to the eligibility criteria and randomly assigned to a low-intensity group (LIG) (40–50% of maximum heart rate), moderate-intensity group (MIG) (60–70% of maximum heart rate) and control group (CG). The exercise protocols consisted of 12 weeks of aerobic exercise (two sessions per week). Psychological well-being and QoL were assessed through the Ryff's Psychological Well-being Scale and the World Health Organization QoL Questionnaire. The statistical analysis for psychological well-being indicated that a significant main group (between-group) (*F* = 11.777, *p* < 0.001, η*p*^2^ = 0.359), time (within-group) (*F* = 58.983, *p* < 0.001, η*p*^2^ = 0.584) and interaction effect (group × time) (*F* = 20.146, *p* < 0.001, η*p*^2^ = 0.490) for PWB total score. Bonferroni *post-hoc* tests revealed that the PWB total score in the MIG group was more significant than both LIG (*p* = 0.003) and CG (*p* < 0.001). Results for PWB components including self-acceptance, positive relationships with others, autonomy, purposeful life, and environmental mastery revealed no significant differences (*p* > 0.05). While there was a significant difference between the groups for personal growth component. Bonferroni *post-hoc* tests revealed that the personal growth in the MIG group was more significant than both LIG (*p* = 0.028) and CG (*p* < 0.001). Result for QoL indicated significant differences for the main group (*F* = 13.277, *p* < 0.001, η*p*^2^ = 0.387), time (*F* = 25.533, *p* < 0.001, η*p*^2^ = 0.378) and interaction effect (*F* = 9.992, *p* < 0.001, η*p*^2^ = 0.332) for QoL total scale. Bonferroni *post-hoc* tests revealed that the QoL total scale in the MIG group was more significant than both LIG (*p* = 0.003) and CG (*p* < 0.001). Results for QoL components including Physical health, Social relationships, Health environment revealed no significant differences (*p* > 0.05), while there was a significant difference between the groups for the Psychological health component. Bonferroni *post-hoc* tests revealed that the Psychological health in the MIG group was more significant than both LIG (*p* = 0.009) and CG (*p* = 0.002). Therefore, aerobic exercise improves PWB and QoL in older adults, moderate-intensity exercise seems to produce higher benefits than low-intensity, demonstrating a positive dose-response relationship.

## Introduction

During the last century, modern societies have faced a demographic change toward an increase in the aging population (1). Advances in medicine, socio-economic improvement, and decreasing fertility have all added to the well-being of mankind and subsequently to changes in the sizes of the different age groups in the population beginning with a decrease in the ratio of young people (2). Over the last 50 years, the number of adults aged 70 and over has tripled; and by 2025–2030 this population will increase 3.5 times, as fast as the general population and it will represent over 25% of the population worldwide (3). Whilst this perspective is one of humankind's chief achievements, it also challenges societies with vast medical tasks (4).

Aging is a gradual, continuous process of natural change that begins in early adulthood. During early middle age, physical and mental functions begin to gradually decline (5). Living longer should be accompanied by good health, active social participation, and safety, but a long lifespan is not necessarily synonymous with good health (6). The World Health Organization (WHO) discovered that 60–85% of people in the world lead sedentary lifestyles, making it one of the most serious community health concerns in western countries (7). Therefore, with such foresight, WHO encourages all health authorities in different countries to increase public awareness in this area and is also pursuing a comprehensive approach to conducting research with common goals in different countries. An inactive lifestyle is increasingly is the main cause of most chronic diseases (8) and has been made worse since the Covid-19 pandemic (9, 10).

Age-induced health problems, for which there are no other clear-cut causative agents, may be better tackled by focusing on health promotion, rather than relying solely on disease management and treatment (11). Persisting in the disease-oriented approach, as opposed to health-oriented and preventive strategies, it is economically, socially, and psychologically unsustainable (12). Variables such as quality of life (QoL), life satisfaction, and psychological well-being (PWB) are of particular importance (13).

In this vein, regular physical activity (PA) can positively effect different aspects of human well-being (14). Well-being reflects life satisfaction, and it is grounded on having positive relationships with others, a sense of personal mastery and autonomy, a feeling of purpose and meaning in life, personal growth and development (15). Psychological well-being is attained through challenging but rewarding life events (16). In this respect, the Six-factor Model of Psychological Well-being was developed by Carol Ryff, inspired by Aristotle's Nicomachean Ethics, “where the goal of life is not feeling good but is instead about living virtuously” (15). In this model, six factors are considered key elements of PWB: Self-acceptance, Personal growth, Purpose in life, Environmental mastery, Autonomy, Positive relations with others.

We contend that PA can play a role in well-being, either directly or indirectly.

Irrespective of age, PA is a vital element of healthy and lively aging (17), and an approach to keep older people socially involved (18). Indeed, older adults are particularly susceptible to become isolated as they face significant life events such as retirement, loved ones becoming unwell or passing away, or moving into care. Hence, social isolation and loneliness are major risk factors that are linked with poor physical and mental health status (19).

Social benefits of PA, such as social interaction with others are often described as factors which contribute to explain its benefits from a transdisciplinary biopsychosocial perspective (20).

The maintenance of high physical function is one of the key factors for successful aging (21, 22). Staying physically and mentally active can therefore not only delay the development of some chronic illnesses and disabilities in older adults, but also improve positive psychological characteristics. In laboratory settings, it has been reported that older adults can improve and increase the positive psychological characteristics by a moderate-intensity exercise program (23). In this sense, one study with representative survey data from 28 European countries, examined the effect of participation intensity and duration on subjective well-being (SWB) and has demonstrated that the number of days people practiced at moderate intensity had a significant and positive effect on SWB, while the number of days with vigorous-intensity activity had a significant and negative impact (24). Thus, the WHO's recommendation (25) of at least 75 min of vigorous-intensity physical activity throughout the week might be effective for physical health, but not for SWB according to the current study. Netz stated also that moderate-intensity activity was the most beneficial activity level to improve mental well-being at an advanced age (26). The relationship between exercise intensity and emotional response can be modeled as an inverted U curve which means that moderate exercise corresponds to optimal emotional change (27). The pleasure obtained from exercise decreases when the intensity of exercise is high, due to older individuals sometimes forgo leisure exercise as an entertainment in the interest of physical fitness by exerting a higher intensity on themselves, and thus experience less pleasure (28).

Still, the laboratory setting cannot provide for all individuals, and easy to implement, naturalistic exercise programs should be attempted.

Therefore, using aerobic exercise in the field setting seems to be necessary for the older individuals. In addition, a recent scoping review highlighted that more emphasis should be placed on studying the elderly in Iran (29, 30). Therefore, the purpose of this study was to examine the effects of low and moderate-intensity aerobic exercises on QoL and PWB in older Iranian people.

Our hypothesis highlights that moderate-intensity PA produces greater benefits than low intensity on QoL and in PWB.

In this regard, various studies have been conducted in different countries in the last decade (14, 31, 32), but due to its necessity, there is an urgent need to do it in different countries due to geographical location or sociocultural and economical status to update and awareness of communities. As a result, WHO, as the main custodian of health in the world, encourages and supports researchers in different countries to do so. This project has been done the first in the Middle East with the support of WHO.

## Materials and Methods

### The Experimental Approach to the Problem

The present study was semi-experimental with pre- and post-test design. We measured the dependent variables before and after the intervention. The research population included male older adults, 65–80 years old, from three regions of Tehran city.

Based on the statistical method analyzed, we estimated power and sample size for the design by F tests ANOVA: Fixed effects, omnibus, one-way; Number of groups = 3; Power (1 – β err prob) = 0.80, and based on a previous studies the effect of exercise on PWB and QoL variables (14, 33–36) that highlighted high to very high effect size. There is an 80.3% (Actual power) chance of correctly rejecting the null hypothesis of no difference in variables of this study results with a total of 42 subjects. For this statistical analysis was used from G-Power software (University of Dusseldorf, Dusseldorf, Germany).

### Participants

Forty-five male adults, aged 65–80 years, were selected according to the eligibility criteria; (a) age between 65 and 80 years, (b) without any psychological problem, (c) not smoking, (d), without history of asthma, respiratory and cardiovascular diseases (e) no taking hypnotic drugs, (f) without any musculoskeletal or fraction problems that would prevent participation in aerobic and physical exercises, (g) not engaged particularly in moderate and vigorous PA. Subjects were then randomly divided into two experimental groups: (low-intensity aerobic exercise (LIG) and moderate-intensity aerobic exercise (MIG) or one control group (CG). Exclusion criteria were as follows: (1) deterioration of health condition; (2) inability to perform training; (3) lack of interest in continuing training; (4) not completing the posttest; and (5) the physician's decision to exclude the participant from the study.

**Figure 1:**
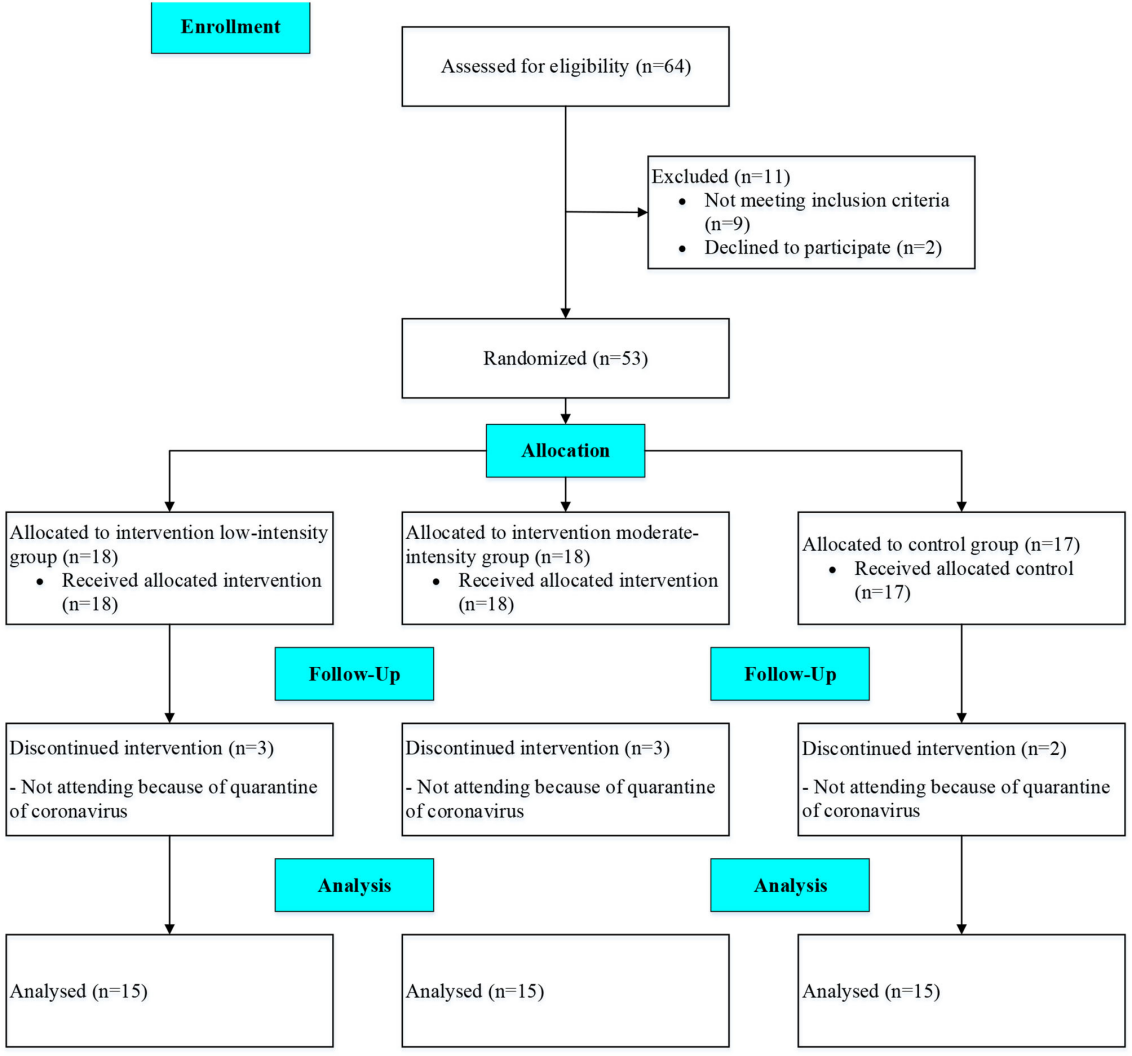
Flow of the study.

The flow of the study is shown in Figure 1.

### Ethics

This study was approved by the Ethics Committee of the Sport Sciences Research Institute of Iran with the cod number of IR.SSRI.REC.1399.954. All subjects received in-formation about the aim and the methodology of the project before the start of the study. Participation was voluntary. All subjects completed the informed consent form before participation. They were assured for: (i) using safe practices during the study; (ii) allowing to withdraw from the study at any time; (iii) protecting their anonymity and confidentiality; and (iv) publishing the results in general. All stages of this study were carried out based on the ethical principles in the Helsinki Declaration.

### Interventions

The exercise protocol consisted of 12-week aerobic-based exercise (2 sessions per week), based on the Rockport one-mile walking/running test. The aerobic exercise sessions were implemented in the mornings (from 8 to 11 am). The exercise program consisted of a 10-min warm-up [20–30% of maximum heart rate (HR_max_)], 35 min of low or moderate aerobic exercises, and a 10-min cool-down. To determine the intensity of exercise for intervention groups, the HR_max_ was estimated by subtracting age from 220 and, based on aerobic exercise type (40–50% HR_max_ for the LIG and 60–70% HR_max_ for the MIG), the target HR_max_ was calculated for each subject (22, 23, 31). The subjects' HR was measured and controlled using chest belts (Polar X-trainer Plus). All training sessions were conducted under the supervision of a trainer in parks.

Participants in the LIG and MIG conducted their exercise protocols, and the CG continued its daily routine activities.

For safety and managing associated risks with aerobic exercises in older adults, all subjects were evaluated by a practitioner to confirm their physical and mental health. Moreover, All the pre- and post-tests were conducted by three expert examiners (37), with expertise in the field of exercise science and motor behavior, they had experience in exercise and work with older adults.

### Data Measurement and Variables

Three questionnaires were used for data collection. In the first step, written per-missions to use the QoL (WHOQOL-26) (38) and PWB questionnaire were obtained from the original authors. Next, the questionnaires were translated into Persian, and after coordinating and unifying the languages (Persian and English), they were translated into English to match the original version. This process was conducted according to the International Quality of Life Assessment (IQOLA) protocol (39). In the next step, the face validity of the questionnaires was determined and the questions with high validity were identified.

To measure PWB, we used the Ryff's scales (15). The original version of this scale contained 120 questions, but in later studies, shorter forms contained 84, 54, and 18 questions. We used the 18-item form in this study (40). This scale contains six sub-scales: self-acceptance, positive relationship with others, autonomy, purposeful life, personal growth, and environmental mastery. In the 18-item form, each factor had 3 points on the Likert scale of 6 points (41). For each question, a score of 1–6 is attributed. Higher scores indicate better PWB.

Moreover, we used the short version of the WHOQoL (WHOQOL-26) to assess the QoL (38). The scale consists of 26 items and four components. Its components include a range of physical, psychological, environmental, and social communication. Trompenaars et al. (42) reported 0.66 and 0.80 for reliability and validity for this scale in the older people. Demographic information such as age, sex, socioeconomic status, educational level, special diseases, surgical history, marital status, number of children of subjects was collected by demographic questionnaire.

### Beneficiaries of Research Results

Regardless of age, PA promotes systemic benefits, including improved cardiovascular health, bone density, and QoL, among many other features (43). And growing evidence demonstrates that PA is beneficial for physical and psychological health in older adults (44), if there is good adherence to the PA programs (45). We believe that our results may provide valuable information not only from the perspective of older adults in general, but also better inform Iranian institutions and potentially assist in the development of better conditions for older adults to engage in PA more regularly.

### Statistical Analysis

Data were analyzed with the Statistical Package of Social Sciences (SPSS, IBM, v22) and presented in mean ± standard deviation (SD). The Kolmogorov–Smirnov test was used to check the normality of the data and the data showed that it was normal. A series of repeated measured analyses of variance (ANOVA) was performed to explore the effect of different interventions on the outcomes. Bonferroni *post-hoc* tests were performed when the differences between conditions were statistically significant. The magnitude of changes was calculated by subtracting post-intervention values from pre-intervention values. Effect sizes (ES) were also computed as the change score divided by the SD of the change score to examine the magnitude of differences while controlling for the influence of the sample size (46) with 0.2 considered as a small ES, 0.2–0.5 as a moderate ES, 0.5–0.8 as a large ES, and > 0.8 as a very large ES (47). The significance level was set at *p* < 0.05 for all statistical analyses.

## Results

### Characteristics of the Participants

Table 1 presents the characteristics of the participants.

**Table 1:** Participants characteristics, mean ± standard deviation.

	**Variables**	**LIG (*N* = 15)**	**MIG (*N* = 15)**	**CG (*N* = 15)**
**Demographic**	**Age (years)**	71.5 ± 3.9	72.2 ± 4.5	74.3 ± 6.3
	**Height (cm)**	172.0 ± 7.4	169.0 ± 6.2	171.0 ± 5.3
	**Weight (kg)**	75.1 ± 4.7	73.3 ± 5.4	73.7 ± 6.5
	**BMI (kg/m** ^**2**^ **)**	25.4 ± 1.3	25.7 ± 1.0	25.2 ± 1.1
**Marital status**	**Married** **Widow** **Divorced** **Single**	11 (24.4%) 3 (6.7%) 0 (0%) 1 (2.2%)	8 (17.8%) 4 (8.9%) 1 (2.2%) 2 (4.5%)	12 (26.7%) 1 (2.2%) 2 (4.5%) 0 (0%)
**Employment**	**Employed** **Not employed** **Second job**	5 (33.3%) 10 (66.7%) 0 (0%)	3 (20.0%) 9 (60.0%) 3 (20.0%)	8 (53.3%) 2 (13.3%) 5 (33.3%)

*BMI, Body Mass Index; LIG, Low Intensity Group; MIG, Moderate Intensity Group; CG, Control Group; ES, Effect Size*.

### Psychological Well-Being (PWB)

Values for psychological well-being are shown in Table 2. The statistical analysis indicated that there was not a significant main group effect (between group) (*F*; 1.42 = 1.207, *p* = 0.309, η*p*^2^ = 0.054), but it was significant for the time (within group) (*F*; 1.42 = 8.442, *p* = 0.006, η*p*^2^ = 0.167) and interaction effect (group × time) (*F*; 1.42 = 4.113, *p* = 0.023, *ηp*^2^ = 0.164) for self−acceptance (Table 2).

**Table 2:** Values for psychological well-being.

**Variable**	**Group**	**Pre**	**Post**	**% Change**	**ES**	p^*^
**Self-acceptance**	LIG	10.1 ± 3.3	11.3 ± 3.6	36.5	0.2	0.309
	MIG	9.2 ± 3.5	13.8 ± 4.1	70.4	1	
	CG	10.0 ± 3.3	10.0 ± 3.2	10.3	0	
**Positive relationships with others**	LIG	7.2 ± 3.1	9.0 ± 3.3	37.4	0.4	0.116
	MIG	8.1 ± 2.1	11.5 ± 3.9	46.5	0.8	
	CG	8.2 ± 3.0	8.4 ± 2.9	12.5	0	
**Autonomy**	LIG	7.2 ± 2.9	8.1 ± 3.1	26.1	0.3	0.177
	MIG	6.9 ± 3.2	10.6 ± 4.2	87.0	0.7	
	CG	7.0 ± 2.9	7.1 ± 2.6	11.3	0	
**Purposeful life**	LIG	5.5 ± 2.7	6.8 ± 2.7	50.9	0.3	0.054
	MIG	5.2 ± 2.3	7.4 ± 3.2	59.9	0.6	
	CG	6.0 ± 3.1	6.1 ± 3.2	17.7	0	
**Personal growth**	LIG	7.1 ± 2.2	9.2 ± 3.3	46.0	0.5	<0.001
	MIG	8.1 ± 3.1	12.4 ± 3.1	76.6	1.0	
	CG	6.8 ± 3.2	7.0 ± 3.2	30.0	0	
**Environmental mastery**	LIG	9.5 ± 3.2	11.3 ± 3.5	26.0	0.7	0.114
	MIG	10.3 ± 2.5	14.1 ± 4.1	40.6	1.1	
	CG	10.4 ± 2.5	10.4 ± 2.5	9.0	0	
**Ryff-18 total scale**	LIG	46.5 ± 8.0	55.8 ± 6.4	21.8	1.5	<0.001
	MIG	47.8 ± 6.3	69.8 ± 10.8	48.2	1.8	
	CG	48.4 ± 7.1	49.0 ± 5.4	3.5	0.1	

* *Repeated measured analyses; LIG, Low Intensity Group; MIG, Moderate Intensity Group; CG, Control Group; ES, Effect Size*.

The statistical analysis indicated there was not a significant main group (*F* = 2.267, *p* = 0.116, η*p*^2^ = 0.097), and interaction effect (*F* = 2.319, *p* = 0.111, η*p*^2^ = 0.099), but it was significant for the time (*F* = 8.750, *p* = 0.005, η*p*^2^ = 0.172) for positive relationships with others.

The statistical analysis indicated there was not a significant main group (*F* = 1.807, *p* = 0.177, η*p*^2^ = 0.079), and interaction effect (*F* = 2.951, *p* = 0.063, η*p*^2^ = 0.123), but it was significant for the time (*F* = 6.273, *p* = 0.016, η*p*^2^ = 0.130) for autonomy.

The statistical analysis indicated there was not a significant main group (*F* = 0.054, *p* = 0.947, η*p*^2^ = 0.003), time (*F* = 3.950, *p* = 0.053, η*p*^2^ = 0.086) and interaction effect (*F* = 1.066, *p* = 0.353, η*p*^2^ = 0.048) for purposeful life.

The statistical analysis indicated there was a significant main group (*F* = 9.772, *p* < 0.001, η*p*^2^ = 0.318), time (*F* = 11.527, *p* = 0.002, η*p*^2^ = 0.215) and interaction effect (*F* = 3.299, *p* = 0.047, η*p*^2^ = 0.136) for personal growth.

The results of Bonferroni *post-hoc* tests revealed that the personal growth in the MIG group were more significant than both LIG (*p* = 0.028) and CG (*p* < 0.001).

The statistical analysis indicated there was not a significant main group (*F* = 2.290, *p* = 0.114, η*p*^2^ = 0.098), but it was significant for the time (*F* = 16.316, *p* < 0.001, η*p*^2^ = 0.280) and interaction effect (*F* = 5.432, *p* = 0.008, η*p*^2^ = 0.206) for environmental mastery.

The statistical analysis indicated there was a significant main group (*F* = 11.777, *p* < 0.001, η*p*^2^ = 0.359), time (*F* = 58.983, *p* < 0.001, η*p*^2^ = 0.584) and interaction effect (*F* = 20.146, *p* < 0.001, η*p*^2^ = 0.490) for PWB total score. The results of Bonferroni *post-hoc* tests revealed that the PWB Total Score in the MIG group were more significant than both LIG (*p* = 0.003) and CG (*p* < 0.001).

### Quality of Life (QoL)

Table 3 shows values for quality of life. The statistical analysis using repeated measures indicated a not significant main group (*F* = 3.181, *p* = 0.052, η*p*^2^ = 0.132), but it was significant for the time (*F* = 13.025, *p* = 0.001, η*p*^2^ = 0.237) and interaction effect (*F* = 4.979, *p* = 0.011, η*p*^2^ = 0.192) for Physical health (Table 3).

**Table 3:** Values for quality of life.

**Variable**	**Group**	**Pre**	**Post**	**% Change**	**ES**	*p* ^*^
**Physical health**	LIG	9.7 ± 3.1	11.3 ± 3.1	27.1	0.4	0.052
	MIG	9.3 ± 3.2	14.0 ± 3.2	69.4	1.1	
	CG	9.2 ± 3.2	9.3 ± 3.8	8.9	0	
**Psychological health**	LIG	7.9 ± 2.4	9.2 ± 3.1	29.6	0.3	0.001
	MIG	8.8 ± 2.3	12.5 ± 3.5	51.8	0.8	
	CG	8.1 ± 2.8	8.2 ± 2.7	14.5	0	
**Social relationships**	LIG	9.7 ± 3.4	11.0 ± 3.2	37.2	0.3	0.140
	MIG	9.3 ± 3.2	13.9 ± 3.7	74.3	0.8	
	CG	10.0 ± 3.5	10.3 ± 3.3	20.3	0.1	
**Health environment**	LIG	8.8 ± 3.7	10.3 ± 2.7	39.7	0.3	0.398
	MIG	8.2 ± 3.5	12.7 ± 4.3	70.8	1.1	
	CG	9.1 ± 3.4	9.1 ± 3.3	12.2	0	
**WHOQOL-26 total scale**	LIG	36.0 ± 7.7	41.9 ± 7.3	21.4	0.5	<0.001
	MIG	35.6 ± 5.0	53.0 ± 6.1	52.6	2.0	
	CG	36.5 ± 5.9	37.0 ± 7.6	6.2	0	

* *Repeated measured analyses; LIG, Low Intensity Group; MIG, Moderate Intensity Group; CG, Control Group; ES, Effect Size*.

Results also indicated a significant main group (*F* = 8.018, *p* = 0.001, η*p*^2^ = 0.276), time (*F* = 7.065, *p* = 0.011, η*p*^2^ = 0.144) and interaction effect (*F* = 2.571, *p* = 0.088, η*p*^2^ = 0.109) for Psychological health. The results of Bonferroni *post-hoc* tests revealed that the Psychological health in the MIG group were more significant than both LIG (*p* = 0.009) and CG (*p* = 0.002).

The statistical analysis using repeated measures indicated a not significant main group (*F* = 2.059, *p* = 0.140, η*p*^2^ = 0.089) and interaction effect (*F* = 2.552, *p* = 0.090, η*p*^2^ = 0.108), but it was significant for the time (*F* = 6.713, *p* = 0.013, η*p*^2^ = 0.138) for social relationships.

Results also indicated a not significant main group (*F* = 0.941, *p* = 0.398, η*p*^2^ = 0.043), but it was significant for the time (*F* = 8.410, *p* = 0.006, η*p*^2^ = 0.167) and interaction effect (*F* = 3.709, *p* = 0.033, η*p*^2^ = 0.150) for Health environment.

The statistical analysis using repeated measures indicated significant differences for main group (*F* = 13.277, *p* < 0.001, η*p*^2^ = 0.387), time (*F* = 25.533, *p* < 0.001, η*p*^2^ = 0.378) and interaction effect (*F* = 9.992, *p* < 0.001, η*p*^2^ = 0.332) for QoL Total scale. The results of Bonferroni *post-hoc* tests revealed that the QoL Total scale in the MIG group were more significant than both LIG (*p* = 0.003) and CG (*p* < 0.001).

### Reliability of the Variables

Cronbach's alpha was used to assess the reliability of the variables of this study (Table 4).

**Table 4:** Reliability of the variables.

**Variable**	**Cronbach's alpha for reliability**
Ryff-18 total score	0.741
Self-acceptance	0.714
Positive relationships with others	0.723
Autonomy	0.701
Purposeful life	0.739
Personal growth	0.801
Environmental mastery	0.765
Quality of life (QoL) total score	0.796
Physical health	0.782
Physical health	0.763
Social relationships	0.825
Health environment	0.812

## Discussion

Despite the growing evidence on the benefits of exercise on older adults' health and functioning, the available literature does not provide robust information concerning the effects of exercise in PWB and QoL in the Islamic Republic of Iran. Moreover, for a deeper understanding and a more personalized implementation, the effect intensity of PA needs to be investigated in more detail. Therefore, the present study aimed at contributing to the scanty literature researching specific dimensions of well-being, based on the Ryff Psychological Well-being Scale (15), and in QoL, in relation to exercise-based interventions addressed among Iranian older people.

The implementation of interventions to promote health and well-being among older citizens is particularly needed, given their increasing number, which is estimated to raise 10.5% in 2025 and 21.7% in 2050 in Iran (48), along with the health benefits associated with exercise in this specific population (25). As age increases, consistent with previous studies in Iran (49), the prevalence of absolute inactivity also increases. In this sense, intervention exercise programs addressed to older citizens are therefore a major goal in the domain of public health (50). Furthermore, there is growing awareness of the need to evaluate their outcomes both, on physical health and at the psychological functioning level.

Based on these premises, this research aimed at evaluating the effects of two intervention exercise programs, through sessions of aerobic exercise with different intensities, addressed to older adults on participants' eudaimonic well-being and in QoL.

In this vein, the most widely used eudaimonic model in clinical interventions is PWB (15), with six dimensions: self-acceptance, mastery of the environment, autonomy, positive relationships, personal growth, and life purposes. Well-being has shown different components, e.g., the comparison among groups revealed differences between the MIG and the LIG and CG in the total score of psychological well-being, as well as in all its components except on purposeful life that presented no difference. One systematic review (44), which investigated the longitudinal relationship between PA and the psychological domains of well-being in older adults, and the possible moderators and mediators of this relationship, demonstrated that leisure time PA (associated with enjoyment, diversion from stress and social connection) at light intensities and conducted in group-settings is the most beneficial for promoting psychological well-being in older adults.

These results are pertinent; however, they differ from ours. It is important to highlight that we identified a great diversity in the applied measuring methods to assess PWB (e.g., Satisfaction with Life Scale; Scale from the Chinese Longitudinal Health Longevity Survey; Positive and negative affect subscales; Louvain Well-Being Scale). In the present study we evaluated PWB by using Carol Ryff Scale. Additionally, some caution is needed in interpreting results, due to the biggest survey which has analyzed PA profile in Iran and that showed that, the largest domain of PA was work (53.7%), transport (33.6%) and recreation (12.8%) (51). Therefore, leisure time PA represents a smaller amount. Thus, the differences in methodology and distinct patterns of PA could explain the different results obtained.

One longitudinal study (52), which examined whether the association between objectively assessed PA (accelerometer) and subjective well-being depended on the exercise intensities (light PA, moderate-to-vigorous PA, sedentary time), indicated that moderate-to-vigorous PA and light PA were linked with different dimensions of well-being, suggesting that different intensities of late-life PA make distinct contributions to well-being. In this previous study, authors assessed subjective well-being through the Chinese Aging Well Profile instead PWB by Carol Ryff Scale. Indeed, there is some variability among the studies regarding how PWB was measured, which makes difficult to compare the results obtained.

The Older People and Active Living study in the UK (53) revealed that moderate-to-vigorous PA had the strongest association with physical well-being but the weakest relationship with mental well-being. Another study, using accelerometry during a 6-month follow-up, also demonstrated that moderate-to-vigorous PA rather than light PA was linked with increased physical health among the older people, but only light PA was related to overall mental health (54). Conversely, another study (26) documented that moderate-intensity PA was the most beneficial for improving mental well-being at an advanced age. Different intensities of exercise may be more beneficial for particular domains of PWB than for others, and a dose-response relationship may not hold for all domains of self-reported well-being. In a study, there were several relevant differences between low and high PA, but not with moderate PA. Moderate PA may probably be better than low PA, however, maybe not enough to generate significant differences (55). Whilst, it is possible that different definitions and thresholds may generate distinct results, something that could be explored in future studies.

A moderator factor could be sedentary time. It is well established that sedentary time is a risk factor independent of PA, i.e., two groups may engage in low PA contexts, but present very distinct sedentary behaviors during their daily businesses (56, 57).

In the present study, moderate-intensity PA produced greater benefits than low-intensity PA, indicating a positive dose-response relationship. Vigorous intensity was not analyzed. The WHO guidelines on PA and sedentary behavior (25) reinforce that the regular practice of a moderate-intensity PA confers benefits such as reduced all-cause mortality, cardiovascular disease mortality, incident hypertension, incident site-specific cancers, incident type-2 diabetes, symptoms of anxiety and depression, and improved cognitive health and sleep. In older adults, PA helps to prevent falls and fall-related injuries, as well as declines in bone health and functional ability (43).

Regarding QoL, the moderate-intensity group had better results in physical health, psychological health and total score domains. Accordingly, one systematic review (58), which included and assessed the association between PA and QoL in older individuals, showed that the evidence was contradictory regarding the dose-response relationship between PA and QoL, and that this relationship could depend on the quality domain evaluated (e.g., the impact of PA intensity on the physical domain of QoL may diverge from its impact on the psychological domain). Consequently, more intervention studies are required to investigate the impact of the intensity of PA on QoL. Our study also highlighted the need to analyze confounding variables, such as the socio-economic context. Despite strong evidence linking physical inactivity to chronic health conditions and increased PA to lower mortality and morbidity in older adults (59), as well as the positive effects of PA on PWB and mental health (49, 50), randomized longitudinal studies are required to further optimize evidence-based exercise recommendations for older adults.

We acknowledge that there are some limitations to this study. First, the study coincided with the pandemic of COVID-19 in Iran, and we lost some of our participants., second, we could not get access to female participants, participants were Iranian citizens, thus belonging to a specific socio-cultural context, which precluded from generalizing results to other countries, characterized by different healthcare and welfare systems, different family organization, and different perspectives on aging. We would be acknowledging that employment status could have played a role and should be better explored in future studies.

## Conclusion

The present study addresses the value of moderate rather than low-intensity PA in enhancing PWB and QoL. Our findings may also provide important information for future public health initiatives to yield tailored exercise recommendations.

## Data Availability Statement

The original contributions presented in the study are included in the article/supplementary material, further inquiries can be directed to the corresponding author/s.

## Ethics Statement

The studies involving human participants were reviewed and approved by Sport Sciences Research Institute of Iran with the cod number of IR.SSRI.REC.1399.954. This study complied with all declarations of Helsinki. The participants provided their written informed consent to participate in this study.

## Author Contributions

HN, AS, JA, and HA: conceptualization. AS, MB, HN, JA, and HA: formal analysis. HN, EM-P, AS, JA, JP-G, and MB: methodology. HN, AS, MB, JA, and LC: software. HN, JA, EM-P, LC, AB, and JP-G: writing—original draft preparation and writing—review and editing. HN, AS, JA, and JP-G: writing—original draft. All authors have read and agreed to the published version of the manuscript.

## Funding

This research was conducted with support of WHO/EMRO as source of funding as RPPH Grant 18-92.

## Conflict of Interest

The authors declare that the research was conducted in the absence of any commercial or financial relationships that could be construed as a potential conflict of interest.

## Publisher's Note

All claims expressed in this article are solely those of the authors and do not necessarily represent those of their affiliated organizations, or those of the publisher, the editors and the reviewers. Any product that may be evaluated in this article, or claim that may be made by its manufacturer, is not guaranteed or endorsed by the publisher.
